# Fallacious and True Theories Concerning Certain Skin Diseases

**Published:** 1888-09

**Authors:** H. Goullon


### FALLACIOUS AND TRUE THEORIES CONCERNING CERTAIN SKIN DISEASES.

Dr. H. Goullon.

(Translated by A. McNeil, M. D., from the *Zeitschrift fur Homœpathie.*)

In "Eckermann's conversations with Goethe," it is stated, in part third, under the date of March 31st, 1839: "Goethe was very sick again lately, so that he could be seen only by his most confidential friends. A few weeks ago he was bled, which was followed by pains in the right leg, until at last his internal disease found vent in a wound on the leg *which was followed by a rapid improvement.*" The expression "wound" is at all events inappropriate, for a wound always presumes a mechanical injury. It must have been a superficial exanthem, else it could not have been followed by the statement "This wound was healed several days ago and he is as cheerful and as gracious as ever." With the appearance of this skin disease, the nature of which is not established, the general health improves. This layman, Eckermann, clearly perceives the casual relation and tacitly acknowledges it.

And how does a part of the present medical world [fortunately a part, not all, as we shall see] judge as to such a connection between internal and external diseases? We only need to read in the various widely read journals. For instance, we meet in the 12th Hft. of the "*Buchs fur Alle.*" in the correspondence of the medical co-laborrators the following advice: "Fourteen days in a horizontal position in bed and during this time bandages wet with lead water [sugar of lead ?] changed every three hours, will certainly cure superficial ulcers of the leg." The allopaths stand ready to cure ulcers of the legs in fourteen days; this sounds well! However, usually, the time of arresting is four weeks, and even then the "open leg" is not always cured; if the lead water could cure the medical counselor of the "*Buchs*" would be a very rich man. And why does not a cure always follow the use of such directions? Because the morbid process is very much deeper than the "superficial ulcer;" the views of these medical men are also very superficial *on these points.*

In the same number of that journal a medical man says that a mild eczema will soon disappear under the use of an ointment of equal parts of zinc and white bismuth in vaseline. But as to what appears in the place of that mild eczema, the medical authority is silent or is in ignorance. Oh! you short-sighted skillful one, who would cure more quickly than the invisible natural power which dwells in every organism. If you succeed, in a seeming cure, you close the safety valve and thereby produce in a short time a catastrophe.

Vaseline, "mineral fat, which does not become rancid," is by no means perfectly innocent and safe. After its use on the knee, I have seen an erysipelatous inflammation of the eyes and also over the entire body an eruption [nettle rash]; at other times it is not well tolerated notwithstanding its pretended innocuous properties.

Chronic ulcers of the legs are often beneficial safety valves, which allow one to live on to old age; one will acknowledge this if he will review some cases. I have known many who have passed eighty years and have had such ulcers for decades. However, if the patients are anxious to have these inconvenient ulcers treated, they must be cured by internal medication. We will soon see with what remedies they are to be treated.

A man came for treatment to the clinic of the University of Utrecht. He had had formerly an ulcerated leg which was healed; after it was closed up he became insane, but reason returned when a seton was made in calf of his leg. This seton he allowed to heal afterward; the mania returned, but *disappeared again on opening the seton afresh.* Can there be a clearer or more convincing proof of the opinion that many times in these cases of eruptions on the skin that the *medicatrix natura*, or the physician of nature, has a definite object in view? I use the word eruption advisedly; for these are real eruptions, volcanoes, of the skin, throwing off the refuse or the materia peccans, of the system. Arsenicum, Silicea, and Sulphur are our most potent remedies for curing chronic ulcers; but Sulphur is particularly useful in all kinds of skin disease. A French physician calls Silicea the first of the anti-scrofulous remedies; and it is acknowledged by both allopath and homœopath that Sulphur corresponds closely to the widespread hæmorrhoidal constitution, whose pathologico-anotomical expression is frequently represented by ulcers. If we remove the fundamental morbid condition by appropriate internal medicine, of which the different affections of the skin are but mere symptoms, then a relapse is almost impossible. Hufeland, the celebrated physician, who had a masterly acquaintance with the art of prolonging life, believed in leaving alone certain forms of eruptions of a scrofulous nature, and would not disturb their growth and natural development. He did nothing to them either by internal or external medication. And we ought to indorse this practice, especially when we see its correctness proven, as in cases of deafness following a suppressed otorrhœa [even from the administration of such homœopathic remedies as Hepar, Mercurius, etc., in too strong doses.]

If any one should go so far as to suppress an eczema around or behind the ears with oxide of zinc ointment and the like, which the allopaths claim to be harmless, and thus drive the disease back on the central organs, then, the outlet for the disease being thus closed, frequently fatal convulsions are produced by this irrational treatment.

There are some learned men who do not deny that eruptions are constitutional, *i. e.*, are to be traced to an origin in some internal general disease; this view, however, does not prevent them from medication which aims at local or suppressive treatment of the disease. Such a partially enlightened man is Professor Nuna, of Hamburg. I was very anxious to learn his position on this question in practical dermatology, in which he is a recognized authority, read his pamplet entitled "*Das Ekzem in Kinkesatter*" [Eczema in Childhood.] The introduction was quite reasonable and encouraging, for he says, "The best paper on this theme is from the pen of Dr. Bulkly, the New York dermatologist. The Doctor sees in eczema [which term here signifies, as it were, eruptives in children] only the expression of a general disease. He does not believe that an eczema can arise from hurtful external influences without such a diathesis. Such eruptions, he thinks, are merely inflammations of the skin. Among the predisposing causes, the trio gout, scrofula, and nervous weakness, play the principal roles. In harmony with these views, the treatment is a combination of external and internal measures. He reports many cures made entirely without any local application; he suspects that the unsuccessful cases of those physicians who rely on local treatment were due to their neglect of constitutional treatment."

Dr. Nuna continues, "Standing on the one-sided local therapie established by Hebra thirty years ago, which has become the fashion, we do not censure this treatment which is brought forward with so much scientific earnestness and which is based on such extensive experience. On the contrary, we congratulate our American colleague on his devotion to his convictions and on the clinical acuteness which he shows in his diagnosis of the constitutions of his patients." Notwithstanding this, Dr. Nuna is afraid to follow the clear sighted teaching of Dr. Bulkly, so he continues to smear his patients *à la Hebra.* The arguments which Dr. Nuna uses to prop up his hybrid views are very fallacious, he says, "Fortunately for humanity they do not always require to be cured in the same way, if they are cured, and we believe that with an especial, but not exclusive local treatment, we have as good results as Bulkly."

But between so-called "curing" and genuine healing, there is a great difference. Allopaths also "cure" intermittents, but they pay no attention to the Quinine cachexia; they also "cure" gout with Salycilic acid, but whether or no disease of the heart remains is another question. They also "cure" syphilis, but the patient suffers from mercurialization, etc., just as after the "curing" of gonorrhœa by astringent injections. The great mass of affections of the glands and internal mucous membranes, stomach, intestines, and lungs, follow after the "curing" [*à la Hebra*] of the different skin diseases.

Homœopathy, as we know, is able to cure in reality all diseases of the skin. I refer here to the beautiful cure of the most obstinate forms of psoriasis with Arsenicum[3], reported in the *Revue Hom. Belge,* and in other cases by internal remedies alone in every potency. Knowing, moreover, the injurious effects of the smearing method of Hebra, we cannot long remain in doubt as to which school of medicine can the better cure diseases of the skin. While Homœopathy possesses in Arsenicum, Graphites, Calcarea, Sulphur, etc., the true curative agents for the cure of an entire army of skin diseases, it is absurd to examine the measures employed by the old school. We find that Nuna mentions oils, liniments, carbo-hydrates, salves of grease and tallow, plasters of farina and vasoline, of dextrine and oxide of zinc, of gum, of lead. He smears his patient with glyceroles cotaining ten grammes of oxide of zinc; he uses also washes, powders, baths, and soaps. To what an irrational point of view a man like Nuna may reach is seen by what we have mentioned of his internal and external medicines for eczema, which he also calls *chemical* remedies. Quicksilver, Iodine, and Sulphur are classed with preparations of tar and ichtyol carbolic acid, napthol, and green soap.

This representative of modern dermatology naïvely sums up his various expedients with the words, "Speaking from the experience of myself and others, I can commend only the following six remedies for internal medication: Calomel, Codliver oil, Arsenic, Sulphate of Lime, Antimony, and Pilocarpin." These remedies are not strangers to us. Arsenic is no more a stranger to us than the Sulphate of Lime, which we know under the more familiar name of Hepar sulphate. It cures many cases of eczematous eruptions and many kinds of ulcers.

The iodine of the codliver oils is not unimportant to us; in its compounds of Kali-iodatum and Arsenicum iodatum, it is a remarkably powerful remedy for phagedenic lupus and ulcers with an acrid corrosive secretion. Ferrum iodatum like Iodine itself is employed with great success. The old school have also employed Iodoform, with, as they claim, success, in ulcers on the legs. Iodoform, like Cocaine and others, are at present fashionable, like some street ballads, and other popular things which come and go—ephemera they are called. It sounds like a joke to mention Calomel among his great specific remedies for eczema, as if there were no better preparations of Quicksilver than Calomel. There is, for example, the red precipitate, Hydrargyrum oxydatum rubrum, which Lutz called the purest preparation of Mercury, and the best adapted for trituration and potentization. Mercurius solubis deserves preference to Calomel.

Let us return to the principal point. We believe firmly in a total condemnation of the theory which prevails in the old school that skin diseases in general and eczema in particular are only of local significance. On the contrary, a part of them, at least, are as inseparable from the organism as the blossom is from the tree. Yea, more, they represent a critical effort of the healing powers of nature, the suppression of which is a defilement of the vitality.

